# FHY1 Mediates Nuclear Import of the Light-Activated Phytochrome A Photoreceptor

**DOI:** 10.1371/journal.pgen.1000143

**Published:** 2008-08-01

**Authors:** Thierry Genoud, Fabian Schweizer, Anke Tscheuschler, Dimitry Debrieux, Jorge J. Casal, Eberhard Schäfer, Andreas Hiltbrunner, Christian Fankhauser

**Affiliations:** 1 Centre for Integrative Genomics, University of Lausanne, Lausanne, Switzerland; 2 Institut für Biologie II/Botanik, Albert Ludwigs Universität, Freiburg, Germany; 3 IFEVA, Facultad de Agronomía, Universidad de Buenos Aires, Buenos Aires, Argentina; 4 Consejo Nacional de Investigaciones Científicas y Técnicas (CONICET), Buenos Aires, Argentina; 5 BIOSS, Centre for Biological Signalling Studies, University of Freiburg, Freiburg, Germany; The University of North Carolina at Chapel Hill, United States of America

## Abstract

The phytochrome (phy) family of photoreceptors is of crucial importance throughout the life cycle of higher plants. Light-induced nuclear import is required for most phytochrome responses. Nuclear accumulation of phyA is dependent on two related proteins called FHY1 (Far-red elongated HYpocotyl 1) and FHL (FHY1 Like), with FHY1 playing the predominant function. The transcription of *FHY1* and *FHL* are controlled by FHY3 (Far-red elongated HYpocotyl 3) and FAR1 (FAr-red impaired Response 1), a related pair of transcription factors, which thus indirectly control phyA nuclear accumulation. FHY1 and FHL preferentially interact with the light-activated form of phyA, but the mechanism by which they enable photoreceptor accumulation in the nucleus remains unsolved. Sequence comparison of numerous FHY1-related proteins indicates that only the NLS located at the N-terminus and the phyA-interaction domain located at the C-terminus are conserved. We demonstrate that these two parts of FHY1 are sufficient for FHY1 function. phyA nuclear accumulation is inhibited in the presence of high levels of FHY1 variants unable to enter the nucleus. Furthermore, nuclear accumulation of phyA becomes light- and FHY1-independent when an NLS sequence is fused to phyA, strongly suggesting that FHY1 mediates nuclear import of light-activated phyA. In accordance with this idea, FHY1 and FHY3 become functionally dispensable in seedlings expressing a constitutively nuclear version of phyA. Our data suggest that the mechanism uncovered in Arabidopsis is conserved in higher plants. Moreover, this mechanism allows us to propose a model explaining why phyA needs a specific nuclear import pathway.

## Author Summary

In response to changes in the environment, animals can take shelter while the sessile plants must adapt to the prevalent conditions. Great plasticity in growth and development are striking examples of how plants cope with a changing environment. In plants, light is both a source of energy and an essential informational cue perceived by several classes of photoreceptors. Phytochrome-mediated light signaling is particularly well studied, because these photoreceptors control all aspects of the plant life cycle. The phytochromes are cytoplasmic in the dark and must enter the nucleus upon light activation to initiate signal transduction. How this important light-regulated event is achieved is poorly understood. Here we describe the function of an evolutionary conserved protein called FHY1 for Far-red elongated HYpocotyl 1. We demonstrate that FHY1 interacts with a light-activated phytochrome in the cytoplasm, allowing the complex to be transported into the nucleus. Interestingly, if this phytochrome can enter the nucleus by another mechanism, FHY1 is no longer required for seedling development, indicating that a major function of FHY1 is to chaperone an activated phytochrome into the nucleus. Our experiments suggest that this mechanism uncovered in Arabidopsis is widely conserved among flowering plants.

## Introduction

Plants are sessile organisms and therefore have to adapt growth and development to the environmental conditions at their site of germination. Light is one of the most important factors directing such adaptive responses and it is involved in many developmental steps throughout the life of plants [1],[2]. To detect intensity, quality (wavelength) and direction of incident light plants have evolved a set of photoreceptors monitoring red/far-red (R/FR), blue/UV-A and UV-B [3]–[7]. The phytochrome family of red/far-red photoreceptors plays a key role in seed germination, leaf and stem development, circadian rhythms, shade avoidance and induction of flowering [8]. Although in higher plants phytochromes are not the primary photoreceptors controlling phototropism and chloroplast movements, the phytochromes modulate these responses [9]–[11].

Phytochromes are homodimeric chromoproteins containing the linear tetrapyrole phytochromobilin as chromophore. They photoconvert between two spectrally distinct forms: the red-light-absorbing Pr and the biologically active far-red light-absorbing Pfr form [3],[12]. As the absorption spectra of the two forms overlap the photoconversion is not complete in either direction. Irradiation with light therefore results in a wavelength-specific equilibrium between the Pr and Pfr forms, with only ∼2% Pfr in far-red light and ∼85% Pfr in red light [13]. Under natural conditions the Pfr/Pr ratio differs dramatically depending on the position of the plant within the community (canopy shade versus open environment) [14],[15].

In Arabidopsis the phytochrome gene family consists of five members (*PHYA*–*E*), among which *PHYA* and *PHYB* play the most prominent functions [16]. phyB is the major red light receptor and mediates the red/far-red reversible low fluence response (LFR). Other members of the phytochrome family contribute to responses primarily controlled by phyB. In contrast, responses to continuous far-red light (high irradiance response, HIR) and to single light pulse of very low fluence light (VLFR) depend exclusively on phyA [1],[3],[12]. Photoreceptor mutants have reduced fitness but only the *phyA* mutant is conditionally lethal, highlighting the importance of this photoreceptor [17],[18]. Its functional importance is further revealed by the high degree of sequence conservation among all angiosperms [19]. phyA is also crucial for the modulation of phototropin responses such as the enhancement of phototropism [10],[11].

The subcellular localization of phytochromes is tightly regulated by light. They localize to the cytosol in the dark but translocate into the nucleus upon light activation, where they interact with several transcription factors (e.g. PIFs, phytochrome interacting factors) [20]–[24]. Given that light-activated phytochromes localize to the nucleus and interact with transcription factors, it is not surprising that 10–20% of the genes in Arabidopsis are subject to regulation by red and/or far-red light [25]. Consequently, nuclear accumulation of the photoreceptor is a key step in both phyA and phyB signaling [26]–[29]. The C-terminal half of phyB presumably contains an Nuclear Localization Signal (NLS), which is masked in the dark by the N-terminal half of the photoreceptor. Light triggers a conformational change, potentially unmasking the NLS and allowing nuclear transport of phyB [30]. This model predicts that the general nuclear import machinery is sufficient for phyB nuclear transport. In contrast, it has recently been shown that nuclear accumulation of phyA depends on two plant specific proteins called FHY1 and FHL [11],[26],[27]. Importantly, these proteins are not required for nuclear accumulation of phyB and for phyB signaling [26],[27]. FHY3 and FAR1, two transposase-related transcription factors, directly control *FHY1* and *FHL* transcription and thus indirectly affect phyA nuclear accumulation [31].

FHY1 and FHL are small proteins (202 and 181 aa, respectively) containing an NLS and a Nuclear Export Sequence (NES) [32],[33]. High similarity between FHY1 and FHL is confined to the 36 most C-terminal amino acids. This small domain is necessary and sufficient for the light-regulated interaction with phyA *in vitro* and it is essential for function *in vivo* [26],[32]. Our previous work has shown that FHY1 and FHL are essential for phyA nuclear accumulation but the molecular mechanism involved remains elusive [26],[27]. Three models can explain the requirement of FHY1/FHL for light-regulated nuclear accumulation of phyA. i) FHY1/FHL may be essential for nuclear import of phyA and work as adapter proteins using their NLS and phyA binding-site to link phyA to the general nuclear import machinery. Alternatively, phyA would enter the nucleus independently of FHY1/FHL but ii) FHY1/FHL action may be required to stabilize phyA and protect it from degradation or iii) to trap it in the nucleus and prevent it from being exported back into the cytosol. In this report we provide strong evidence for a model, in which FHY1 and FHL work as adaptor proteins facilitating nuclear transport of phyA. Our data reveal an intriguing system for regulated nuclear transport of a cargo protein that does not contain an NLS of its own.

## Results

### The NLS and the phyA-Interaction Domain Are the Only Functionally Important Parts of FHY1

**Figure 1:**
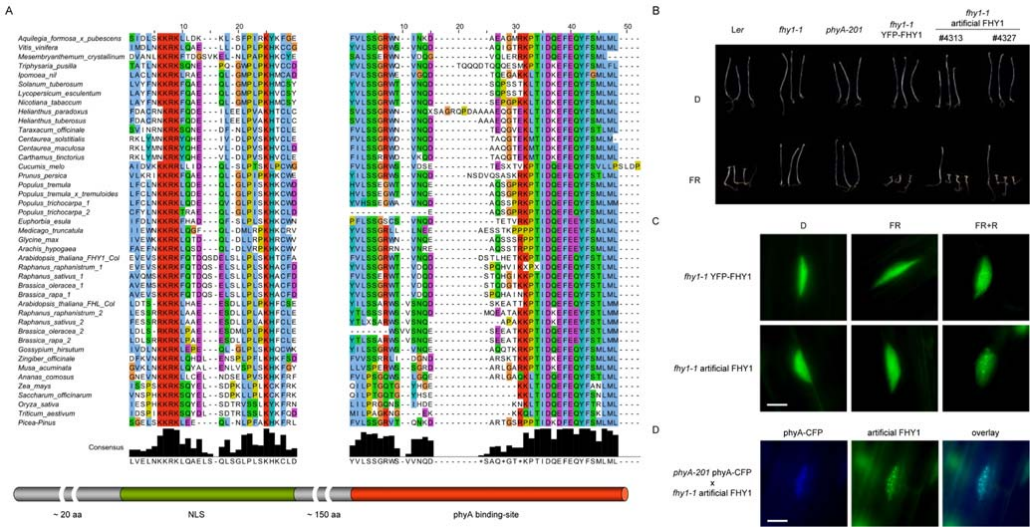
An artificial FHY1 complements the *fhy1* mutant phenotype. (A) Sequence alignment for FHY1-like proteins. The C-terminal 36 aa of Arabidopsis FHY1 were used as a query to search genomic and EST databases. Part of the sequences were assembled from overlapping EST clones. The alignment was done using MAFFT v6.240 (http://align.bmr.kyushu-u.ac.jp/mafft/software/) and Jalview [65]. The sequence Picea-Pinus was derived from EST clones of *Picea glauca* and *Pinus taeda*. At the bottom of the alignment the consensus sequence is shown. The accession numbers of the clones used for the alignment are listed in Table SI. (B) An artificial FHY1 complements the *fhy1* mutant. Wild-type (L*er*), *fhy1*-*1*, and *phyA*-*201* seedlings as well as lines expressing either *P_35S_*∶*YFP*-*FHY1* or *P_35S_*∶*NLS*-*YFP*-*FHY1 167*–*202* (artificial FHY1) in *fhy1*-*1* background were grown for 5 days in the dark or in weak far-red light (0.9 µmol m^−2^ s^−1^). #4313 and #4327 are independent T2 lines segregating into non-transgenic (*fhy1*-*1*) and transgenic (*fhy1*-*1* artificial FHY1) individuals. (C) Artificial FHY1 behaves like native Arabidopsis FHY1. 3-day-old dark-grown *fhy1*-*1* seedlings complemented with either *P_35S_*∶*YFP*-*FHY1* or *P_35S_*∶*NLS*-*YFP*-*FHY1 167*–*202* (artificial FHY1) were used for fluorescence microscopy. The seedlings were analyzed directly (D) or irradiated for 7 h with far-red light, either followed by a 1 min red light pulse (FR+R) or not (FR) prior to microscopic analysis. The scale bar represents 10 µm. (D) Artificial FHY1 colocalizes with phyA. *fhy1*-*1 P_35S_*∶*NLS*-*YFP*-*FHY1 167*–*202* was crossed into *phyA*-*201 P_PHYA_*∶*PHYA*-*CFP*. F1 seedlings were grown for 3 days in the dark, irradiated for 6 h with FR (15 µmol m^−2^ s^−1^) and used for microscopic analysis. The scale bar represents 10 µm.

The high degree of sequence conservation among phyA in angiosperms suggests that the same might be true for phyA signaling components, such as FHY1 and FHL [19]. Yet, the amino acid identity between them is below 30% although they are functional homologs [33]. The only motifs conserved in FHY1 and FHL are the NLS (and to a minor degree the NES) in their N-terminal region and the phyA binding-site at the C-terminus. A database search for FHY1/FHL homologs revealed the presence of FHY1-like proteins in numerous plant species. This is interesting given the key function of FHY1/FHL in phyA signaling in Arabidopsis. The only motifs conserved between all the FHY1-like proteins found in the database and Arabidopsis FHY1/FHL are the NLS and the C-terminal phyA binding-site (Figure 1A). In contrast, the ∼150 aa linking the NLS and the motif essential for interaction with phyA are too diverse to be aligned. Together with the finding that the FHY1/FHL homologs from both rice and dandelion complement the *fhy1* mutant phenotype (data not shown) this suggests that FHY1-like proteins may be defined as proteins containing an NLS and an “FHY1 type” phyA binding-site separated by a ∼150 aa spacer. To test whether this definition holds true we generated an artificial FHY1 consisting of an SV40 NLS and the C-terminal 36 aa of Arabidopsis FHY1 (FHY1 167–202 = FHY1 CT) with Yellow Fluorescent Protein (YFP) as a spacer in between. *fhy1* mutant seedlings expressing this artificial FHY1 under the control of the CaMV 35S promoter were hypersensitive to FR, similar to *fhy1* seedlings complemented with *P_35S_*∶*YFP*-*FHY1* (Figure 1B). Furthermore, the artificial FHY1 accumulated in the nucleus and colocalized with phyA in light-induced nuclear speckles (Figure 1C, D) thus behaving like Arabidopsis FHY1/FHL [26],[27]. We therefore conclude that the NLS and the phyA binding-site of FHY1/FHL are necessary and sufficient for phyA nuclear accumulation.

### A Constitutively Nuclear-Localized phyA Efficiently Rescues a *phyA* Mutant

**Figure 2:**
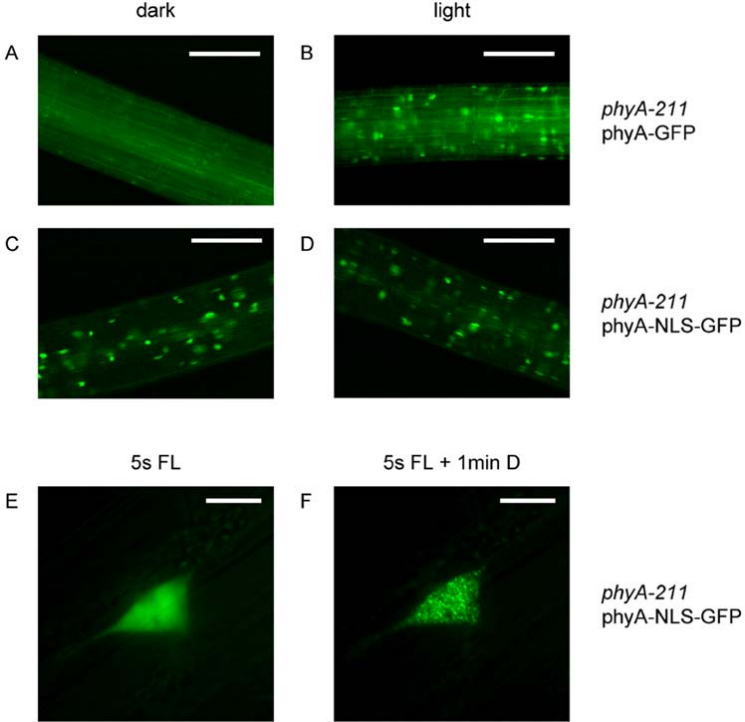
Subcellular localization of a constitutively localized phyA (phyA-NLS-GFP). (A)–(D) 3-day-old dark-grown *phyA*-*211* seedlings complemented with either *P_PHYA_*∶*PHYA*-*GFP* or *P_PHYA_*∶*PHYA*-*NLS*-*GFP* were analyzed by fluorescence microscopy. The seedlings were analyzed directly (dark) or after 10 min irradiation with white light. The scale bars represent 250 µm. (E) and (F). 4-day-old dark-grown *phyA*-*211* seedlings complemented with *P_PHYA_*∶*PHYA*-*NLS*-*GFP* were analyzed by fluorescence microscopy. The preparation of the seedlings and the adjustment of the focal plane were done in safe green light. Then the fluorescence light (FL) was switched on for 5 s and a picture was taken (E). After 1 min incubation in the dark another picture was taken (F). The scale bars represent 10 µm.

Given that both the NLS and the phyA-interaction domain of FHY1 are sufficient for FHY1 activity we tested whether adding the NLS to phyA directly would be enough to promote nuclear localization of phyA fused to the Green Fluorescent Protein (GFP). *phyA* null mutants transformed with either *PHYA*-*GFP* (Figure 2A, B) or *PHYA*-*NLS*-*GFP* (Figure 2C–2F) driven by the *PHYA* promoter were analyzed microscopically. As previously described [23] nuclear accumulation of phyA-GFP was light-dependent (Figure 2A, B). In contrast, in lines expressing phyA-NLS-GFP nuclear localization was constitutive (Figure 2C, D). Nuclear bodies appeared extremely rapidly upon light excitation in phyA-NLS-GFP plants. When nuclei of etiolated phyA-NLS-GFP seedlings were imaged without a light treatment or immediately after a 5 sec red light pulse a smooth nucleoplasmic staining was observed (Figure 2E, data not shown). However as little as 1 minute after a 5 sec red light pulse nuclear bodies appeared in those nuclei (Figure 2F).

**Figure 3:**
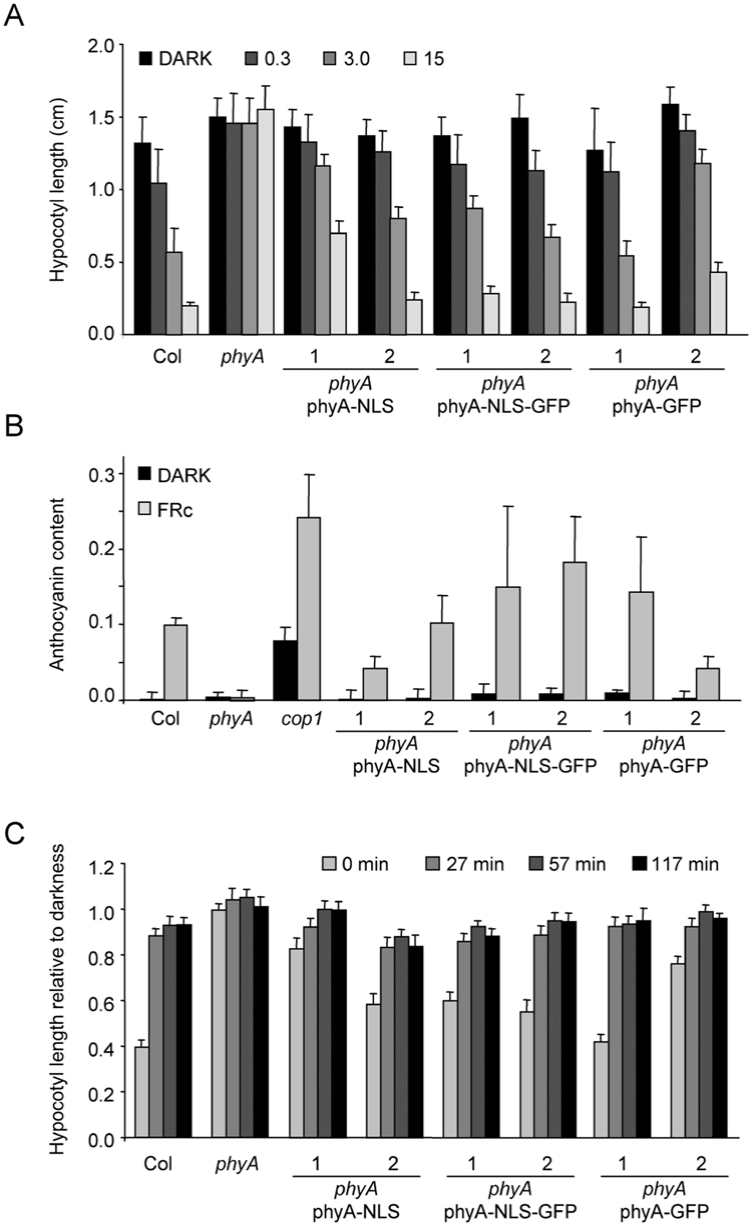
A constitutively localized phyA is functional but does not trigger constitutive photomorphogenesis. (A) FR-HIR for inhibition of hypocotyl elongation. Col, *phyA*-*211* and *phyA*-*211* seedlings expressing phyA-NLS, phyA-NLS-GFP or phyA-GFP (two independent lines each) were grown in the dark (D) or in FR (0.3, 3 or 15 µmol m^−2^ s^−1^). After 5 days the hypocotyl length was measured. The mean value and the SD are indicated with n>15. (B) FR-HIR for anthocyanin accumulation. Col, *phyA*-*211* and *cop1*-*4* as well as the transgenic lines described in (A) were grown in the dark or in FR (5 µmol m^−2^ s^−1^). After 4 days the anthocyanin content was measured. The mean value (A530–A647/seedling) of three replicates and the SD are indicated. (C) VLFR for inhibition of hypocotyl elongation. Col, *phyA*-*211* as well as the lines described in (A) were grown for one day in the dark and then exposed for 3 days to either continuous FR (20 µmol m^−2^ s^−1^) or 3 min FR pulses (20 µmol m^−2^ s^−1^) with different dark intervals (27, 57 and 127 min). At the end of the FR treatment the hypocotyl length was measured. Error bars indicate the SEM (n = 11).

The phenotypic consequences of expressing a constitutively nuclear version of phyA was evaluated by comparing wild type, *phyA* and *phyA* transformed either with a construct encoding *PHYA*-*GFP*, *PHYA*-*NLS*-*GFP* or *PHYA*-*NLS*. Western blot analysis of dozens of independent transgenics showed that while we obtained lines expressing wild-type levels of phyA-GFP at a reasonable frequency (10–20%) we never found lines expressing high levels of either phyA-NLS or phyA-NLS-GFP (data not shown). For our phenotypic analysis we used two homozygous single insertion lines for each construct. phyA-GFP line 1 expressed wild-type levels of phyA while phyA-GFP line 2 expressed phyA levels comparable to the highest expressing phyA-NLS-GFP lines we obtained (Figure S1). Despite the relatively low levels of phyA, the phyA-NLS-GFP lines rescued the FR-HIR phenotype of *phyA* mutants very efficiently for hypocotyl elongation and anthocyanin accumulation (Figure 3A, B). Moreover, the phyA-NLS and phyA-NLS-GFP lines also showed a normal phyA-mediated VLFR response for inhibition of hypocotyl elongation in response to pulses of FR light (Figure 3C). It should also be noted that, despite having constitutively nuclear phyA, phyA-NLS (-GFP) lines did not show a *cop* (constitutively photomorphogenic) phenotype, indicating that nuclear import of phyA is not sufficient to trigger a light response (Figure 3 and data not shown).

### FHY1 Is Dispensable in Plants Expressing a Constitutively Nuclear phyA

**Figure 4:**
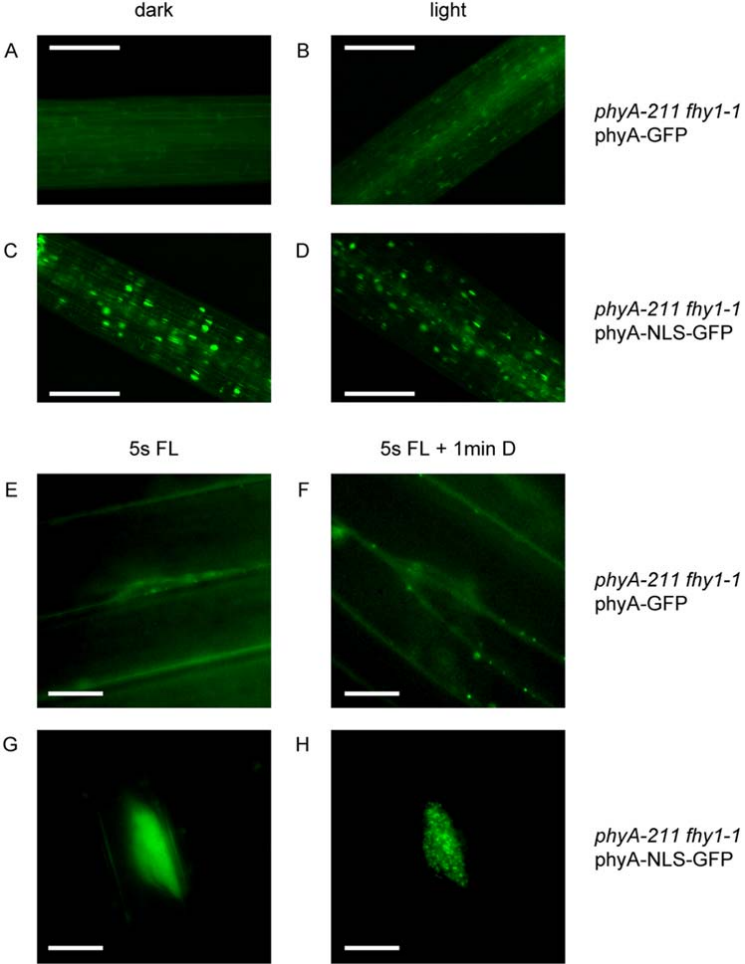
The subcellular localization of a constitutively localized phyA is not dependent on FHY1. (A)–(D) 3-day-old dark-grown *phyA*-*211 fhy1*-*1* seedlings expressing either *P_PHYA_*∶*PHYA*-*GFP* or *P_PHYA_*∶*PHYA*-*NLS*-*GFP* were analyzed by fluorescence microscopy. The seedlings were analyzed directly (dark) or after 10 min irradiation with white light. The scale bars represent 250 µm. (E)–(H) 4-day-old dark-grown *phyA*-*211 fhy1*-*1* seedlings expressing either phyA-GFP (E, F) or phyA-NLS-GFP (G, H) were analyzed by fluorescence microscopy. The preparation of the seedlings and the adjustment of the focal plane were done in safe green light. Then the fluorescence light (FL) was switched on for 5 s and a picture was taken (E and G). After 1 min incubation in the dark another picture was taken (F and H). The scale bars represent 10 µm. (A, B, E, F) *phyA*-*211 fhy1*-*1 P_PHYA_*∶*PHYA*-*GFP* (Col×L*er*) (C, D, G, H) *phyA*-*211 fhy1*-*1 P_PHYA_*∶*PHYA*-*NLS*-*GFP* (Col×L*er*).

The nuclear localization of phyA-NLS-GFP in darkness (Figure 2C), a condition, where there is much reduced phyA-FHY1 interaction, suggested that phyA-NLS-GFP nuclear accumulation did not require FHY1. In order to test this hypothesis genetically we crossed *phyA* phyA-NLS-GFP with *fhy1* mutants and selected siblings in the F2 that were homozygous for *phyA*, *fhy1* and the transgene. Microscopic analysis of such seedlings demonstrated that neither nuclear accumulation nor light induced formation of nuclear bodies of phyA-NLS-GFP required FHY1 (Figure 4C, D, G, H). In control experiments we confirmed that for phyA-GFP plants light-dependent nuclear import was strongly dependent on FHY1 (Figure 4A, B, E, F and data not shown) [26],[27]. We concentrated our analysis on *fhy1* mutants because *fhy1* has a much stronger phenotype than *fhl* [33].

**Figure 5:**
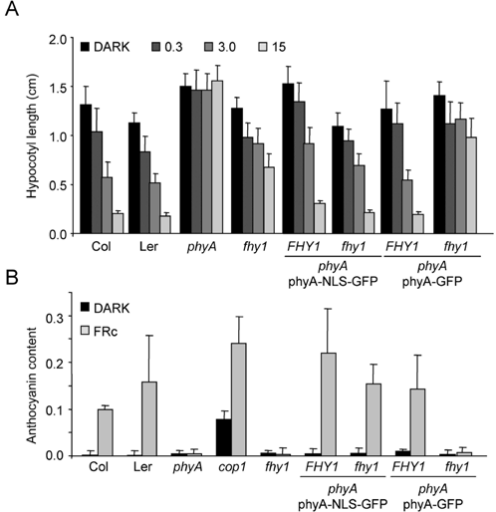
A constitutively nuclear localized phyA can compensate for the absence of FHY1. (A) FR-HIR for inhibition of hypocotyl elongation. *phyA*-*211 P_PHYA_*∶*PHYA*-*GFP* and *P_PHYA_*∶*PHYA*-*NLS*-*GFP* were crossed into *fhy1*-*1*. In the F2 generation seedlings homozygous for the transgene and the *phyA*-*211* mutation and either wild-type (*FHY1*) or homozygous *fhy1*-*1* at the *FHY1* locus were selected. Col, L*er*, *phyA*-*211* and *fhy1*-*1* seedlings as well as *phyA*-*211* seedlings expressing phyA-NLS-GFP or phyA-GFP in *FHY1* and *fhy1*-*1* background were grown in the dark (D) or in FR (0.3, 3 or 15 µmol m^−2^ s^−1^). After 5 days the hypocotyl length was measured. The mean value and the SD are indicated with n>15. (B) FR-HIR for anthocyanin accumulation. The same seedlings as described in (A) as well as the *cop1*-*4* mutant were grown in the dark or in FR (5 µmol m^−2^ s^−1^). After 4 days the anthocyanin content was measured. The mean value (A530–A647/seedling) of three replicates and the SD are indicated.

Given that nuclear accumulation of phyA-NLS-GFP did no longer require FHY1, we tested whether *fhy1* mutants expressing phyA-NLS-GFP had a normal light response to continuous FR light. Interestingly, both the hypocotyl elongation and anthocyanin accumulation phenotypes of *fhy1* mutants were efficiently rescued by phyA-NLS-GFP but not by phyA-GFP (Figure 5). Our data thus indicate that FHY1 becomes dispensable in seedlings expressing phyA-NLS-GFP, suggesting that during the FR-HIR FHY1 is only necessary to control nuclear accumulation of phyA.

**Figure 6:**
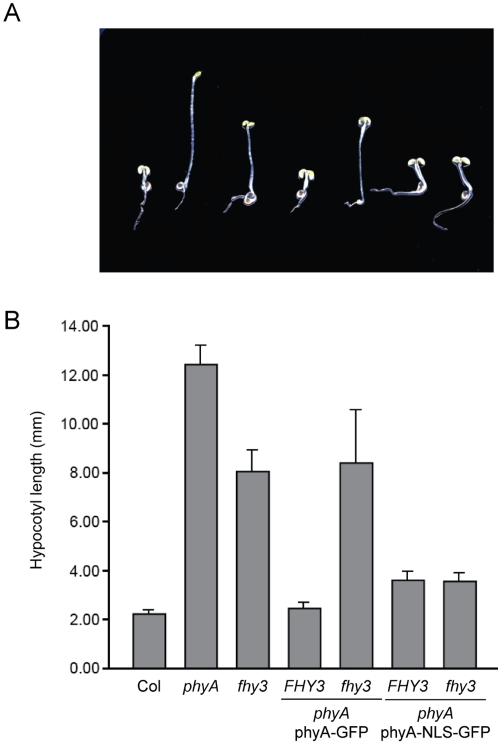
A constitutively nuclear localized phyA can compensate for the absence of FHY3. (A) Morphology of seedlings grown for 5 days in continuous FR (15 µmol m^−2^ s^−1^) light. (B) FR-HIR for inhibition of hypocotyl elongation. Col, *phyA*-*211* and *fhy3*-*1* seedlings as well as *phyA*-*211* seedlings expressing phyA-NLS-GFP or phyA-GFP in *FHY3* and *fhy3*-*1* background were grown as in (A). The mean value and the SD are indicated with n>15.

It was recently shown that FHY3 and FAR1, two closely related transcription factors, directly regulate the expression of *FHY1* and *FHL* [31]. Given that phyA-NLS-GFP could rescue the *fhy1* phenotype, we hypothesized that this construct may also be capable of rescuing *fhy3* mutants, in which the major defect appears to be reduced *FHY1* and *FHL* levels. We restricted our analysis to *fhy3* mutants because FHY3 plays a significantly more important role for this response than FAR1 [31],[34]. We thus crossed *fhy3* with phyA-NLS-GFP plants and analyzed homozygous wild type and mutant *fhy3* siblings. Our phenotypic characterization of the response to far-red light showed that while phyA-NLS-GFP rescued the *fhy3* mutant phenotype phyA-GFP could not (Figure 6). Our results are thus consistent with the notion that the major function of FHY1 and FHY3 is to respectively operate a directly and indirectly control of phyA nuclear accumulation.

### FHY1 Is Important for Nuclear Import of phyA

The only functionally important and widely conserved parts of FHY1 are the NLS and the phyA-interaction domain (Figure 1) [26],[32],[33]. Moreover, nuclear accumulation of phyA-NLS-GFP occurred independently of light and FHY1 (Figures 2 and 4). Taken together these data support the notion that FHY1 mediates light-dependent nuclear import of phyA upon interaction in the cytoplasm. A prediction of this model is that over-expression of either native or artificial FHY1 lacking the NLS should sequester phyA in the cytoplasm and thus result in a dominant negative phenotype.

**Figure 7:**
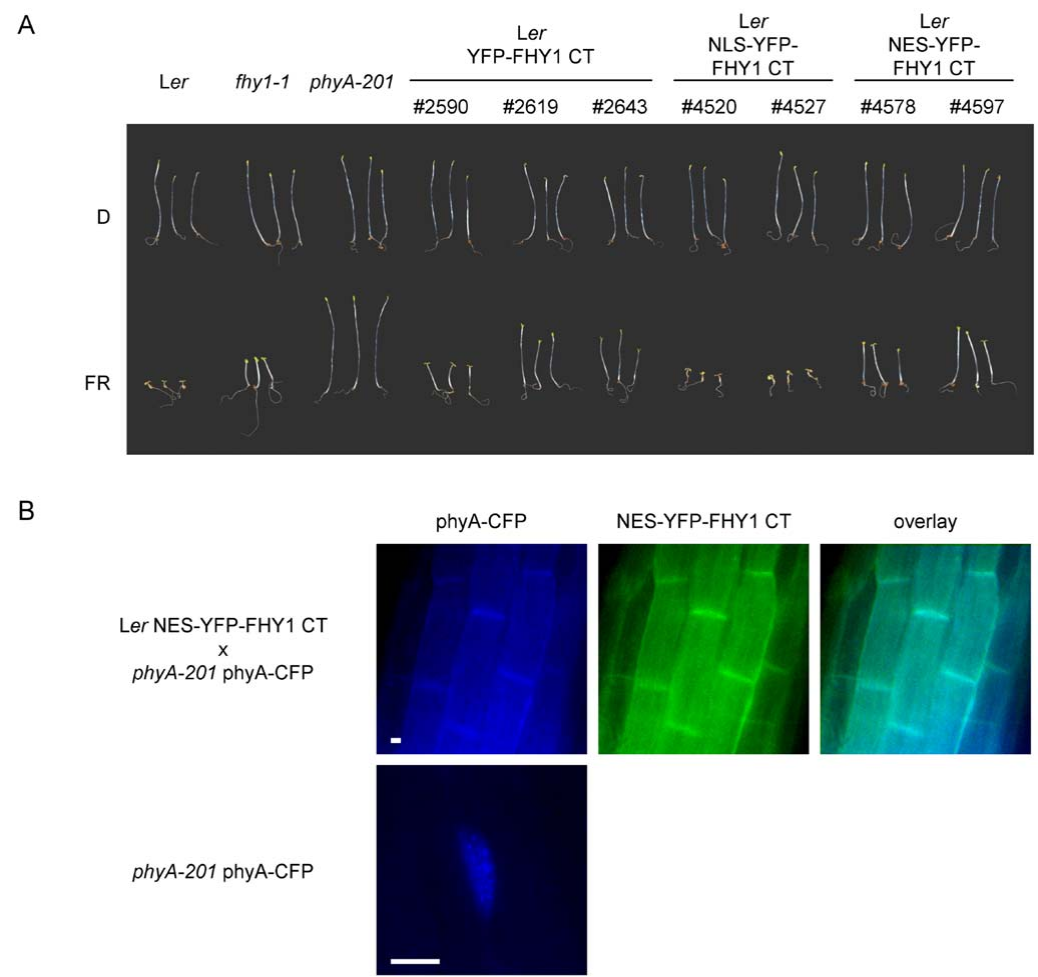
Cytoplasmically localized FHY1 CT induces a dominant negative phenotype. (A) Morphology of seedlings expressing FHY1 CT. Wild-type (L*er*), *fhy1*-*1*, and *phyA*-*201* seedlings as well as transgenic lines expressing different FHY1 167–202 ( = FHY1 CT) constructs in wild-type background were grown for 5 days in the dark or in FR (15 µmol m^−2^ s^−1^). #2590, #2619, #2643; L*er P_35S_*∶*YFP*-*FHY1 167*–*202* (L*er* YFP-FHY1 CT) #4520, #4527; L*er P_35S_*∶*NLS*-*YFP*-*FHY1 167*–*202* (L*er* NLS-YFP-FHY1 CT, i.e. artificial FHY1) #4578, #4597; L*er P_35S_*∶*NES*-*YFP*-*FHY1 167*–*202* (L*er* NES-YFP-FHY1 CT) (B) Cytoplasmically localized FHY1 CT inhibits phyA nuclear accumulation. L*er P_35S_*∶*NES*-*YFP*-*FHY1 167*–*202* was crossed into *phyA*-*201 P_PHYA_*∶*PHYA*-*CFP*. F1 seedlings were grown for 3 days in the dark, irradiated for 6 h with FR (15 µmol m^−2^ s^−1^) and used for microscopic analysis. The scale bars represent 10 µm.

To test this hypothesis we omitted the SV40 NLS in the artificial FHY1 or replaced it by an NES and transformed the constructs (i.e. (NES-) YFP-FHY1 CT) into wild-type plants. As the fusion proteins encoded by the constructs are below the size exclusion limit of the nuclear pore [35] they can enter the nucleus by diffusion but do not accumulate there due to the absence of an NLS. The NES containing version, which is predicted to be actively exported from the nucleus, localized mainly to the cytosol (Figure 7B). As predicted by the nuclear import model, seedlings expressing these constructs were strongly hyposensitive to FR (Figure 7A). This phenotype is consistent with the previous finding that FHY1 containing a disrupted NLS does not complement the *fhy1* phenotype but rather results in an almost complete loss of FR sensitivity [32]. Western blot analysis confirmed that the phyA levels were normal in seedlings expressing (NES-) YFP-FHY1 CT thus excluding the possibility that reduced amounts of phyA were responsible for the dominant negative phenotype (Figure S2). However, NES-YFP-FHY1 CT strongly inhibited phyA nuclear accumulation when crossed into plants expressing Cyan Fluorescent Protein (CFP) tagged phyA (Figure 7B). This suggests that in the cytosol NES-YFP-FHY1 CT competes with endogenous FHY1/FHL for binding to phyA (-CFP) and thereby interferes with phyA (-CFP) nuclear transport.

## Discussion

It was previously shown that FHY1 and its paralogue FHL are required for nuclear accumulation of phyA [26],[27]. The analysis of mutants clearly demonstrates that FHY1 plays the predominant function for both phyA nuclear accumulation and phyA-mediated light responses [27],[33]. This is presumably due to the roughly 15-fold higher level of *FHY1* mRNA compared to *FHL* [33]. We therefore restricted our analysis to the *fhy1* single mutant background, i.e. in the presence of functional FHL. Both FHY1 and FHL interact with light-activated phyA through a conserved C-terminal domain [26]. However, the mechanism, by which these proteins enable nuclear localization of phyA, remains to be established. Our phylogenetic analysis shows that, similarly to phyA, FHY1-related proteins are widely distributed among angiosperms (Figure 1A), suggesting conservation of this aspect of phyA signaling. Moreover, this analysis shows that among FHY1-like proteins only the amino-terminal NLS, which is essential for the interaction with importin alpha (Figure S3), and the carboxy-terminal phyA-interaction domain are conserved. It has previously been shown that both these domains of FHY1 are necessary for function [32]. Our analyses now show that they are also sufficient for FHY1 activity and that the ∼150 aa in between do not perform an essential function. The simplest model (hereafter termed “import” model) accounting for those results is that upon light excitation phyA interacts with FHY1 in the cytoplasm and that this complex enters the nucleus using the general nuclear import machinery (Figure S6).

According to this model adding a strong (and exposed) NLS to phyA should render phyA nuclear accumulation both light- and FHY1-independent. Our experiments show that these predictions are fulfilled in plants expressing phyA-NLS-GFP (Figures 2 and 4). In addition, when an FHY1 variant lacking the NLS sequence is over-expressed in wild-type plants this construct sequesters phyA in the cytoplasm and results in a dominant-negative de-etiolation phenotype (Figure 7). These observations are fully consistent with the notion that FHY1 mediates light-regulated phyA nuclear import by binding selectively to the active Pfr form of phyA in the cytosol and, thereby, linking phyA in a regulated manner to the nuclear import machinery (Figure S6). Our findings indicate that during de-etiolation in far-red light the system essential for nuclear localization of phyA, i.e. FHY3 and FHY1, can be replaced by simply attaching an NLS to phyA. It is however highly unlikely that such plants do not show a decrease in fitness under more natural conditions. The complex system relying on FHY3/FAR1 and FHY1/FHL is highly conserved in evolution (Figure 1A) [31] and FHY1-like proteins from dandelion and rice can compensate for the absence of FHY1 in Arabidopsis (data not shown). The strict conservation of FHY1-like proteins in angiosperms (in the sense of proteins containing a phyA binding-site linked to an NLS) points to a common molecular mechanism of phyA nuclear import and underlines the importance for regulated subcellular localization of phyA. An obvious advantage of the FHY1/phyA system over targeting phyA to the nucleus using an NLS is that it allows for co-existence of nuclear and cytosolic phyA pools and that the pool sizes can be regulated. This may be especially important with regard to possible cytosolic functions of phytochromes as recently described [11]. Nuclear import of phyB does not rely on the FHY1/FHY3 pathway but is light regulated nevertheless [23],[26],[27],[36],[37]. The FHY1-mediated nuclear import described here may explain how phyA can be imported so rapidly in response to light and how this import is possible under light conditions where the pool of Pfr is extremely small [13]. Such conditions are typically encountered for phyA-controlled light responses, such as the VLFR and the FR-HIR [1].

Two alternative scenarios for FHY1 function have been proposed, in which nuclear transport of phyA would not depend on FHY1-like proteins and may even be light-independent (i.e. both Pr and Pfr are transported) [26]. In these models (hereafter referred to as the “FHY1 nuclear anchor” and “protection” models) phyA could either be trapped in the nucleus or protected from degradation by binding to FHY1. As the phyA/FHY1 interaction is light dependent, these models would explain the light regulated nuclear accumulation of phyA as well. Yet, these hypotheses are inconsistent with our data for several reasons. In etiolated seedlings phyA protein levels are much higher than FHY1 (data not shown). This renders both the “FHY1 nuclear anchor” and the “protection” models difficult to envisage unless one FHY1 molecule would bind to multiple phyA proteins. In the “import” model one FHY1 molecule would transport one phyA dimer *per cycle* resulting in nuclear accumulation of large numbers of phyA molecules after multiple transport cycles. In addition, the subcellular localization of phyA-NLS-GFP was not affected in the *fhy1* mutant background (Figure 4), which is only compatible with the “nuclear import” model. The normal localization of phyA-NLS-GFP in *fhy1* mutants is also supported functionally, given that this construct complements *fhy1* (Figure 5). Moreover, western blot analyses show that FHY1 does not affect phyA protein levels in far-red light irrespective of whether phyA enters the nucleus using FHY1 [11]. Moreover the abundance of constitutively nuclear phyA-GFP was also unaffected in the *fhy1* background (Figure S4). These data indicate that FHY1 does not act by protecting phyA from degradation once the photoreceptor entered the nucleus.

Although phyA strongly accumulates in the nucleus in response to irradiation with FR *in vivo* spectroscopic measurements indicate that not significantly more than ∼2% of the total phyA is in the Pfr form under such conditions [38]. This strongly suggests that in FRc the major fraction of nuclear phyA is in the Pr and not the Pfr form [12]. Furthermore, yeast two hybrid experiments show that the light-induced interaction of FHY1 and phyA is R/FR reversible, suggesting that the phyA/FHY1 complex rapidly dissociates upon conversion of Pfr to Pr (Figure S5). It is, however, inherent to the “FHY1 nuclear anchor” and “protection” models that FHY1 has to be bound to phyA to inhibit its export into the cytosol or protect it from degradation. Again, the only model compatible with our findings is the “import” model, where an interaction for a limited time period would be sufficient to allow accumulation of phyA in the nucleus. A constitutive interaction of phyA and FHY1 may even interfere with phyA nuclear accumulation as it may block recycling of FHY1. Once in the nucleus phyA would be trapped in the “import” model – irrespective of whether it is in the Pr or Pfr form – because it is too big to exit the nucleus by diffusion. Taken together our findings strongly support the import model (Figure S6).

After accumulation in the nucleus phyA interacts with various transcription factors (e.g. PIFs) [20],[21],[24]. It is noteworthy that nuclear body formation is still light dependent for phyA-NLS-GFP (Figure 2). Moreover, formation of these subnuclear structures does not require FHY1 (Figure 4) although FHY1 and phyA have been found in light-induced nuclear bodies (Figure 1) [11],[26],[27]. The light-induced nuclear bodies may thus represent sites of phyA-PIF interaction as has previously been reported [39],[40]. Complementation of the *fhy1* mutant by phyA-NLS (-GFP) shows that the interaction of phyA and downstream signaling components does not require FHY1. Rather, binding of FHY1 may prevent the interaction of phyA and effectors. If dissociation of the phyA/FHY1 complex were a prerequisite to initiate downstream signaling this would be an additional argument against the “FHY1 nuclear anchor” and “protection” models. Answering these questions will provide a “molecular” link between phyA nuclear accumulation and initiation of the signaling cascade(s) leading to transcriptional regulation of 10–20% of the genes in the Arabidopsis genome [25],[41],[42].

Adding a strong NLS to phyA results in light- and FHY1-independent nuclear accumulation of the protein. Nevertheless, dark-grown seedlings expressing such constitutively nuclear localized phyA display a normal morphology in darkness and still show normal light responses (phyA-mediated VLFR and HIR) (Figure 3). The fluence-rate dependency and the need for sustained excitation are hallmarks of the HIR [1] and it is well established that nuclear accumulation *per se* is an HIR [23],[43]. Yet, maximal hypocotyl growth inhibition and anthocyanin accumulation in seedlings expressing constitutively nuclear localized phyA are still fluence-rate dependent and require continuous irradiation (Figure 3). Thus, the “physiological HIR” does not derive exclusively from the HIR characteristics of phyA nuclear accumulation, indicating that in wild-type plants more than only one step in phyA signaling is an HIR. The phenotype of plants expressing constitutively nuclear phyA is thus clearly distinct to the partial *det*/*cop* phenotype of a mutant expressing a constitutively Pfr-like phyA [44]. Thus, control of phyA nuclear accumulation does not seem to play an essential role to prevent initiation of downstream signaling in the absence of light, which is crucial for the highly sensitive VLFR. The different affinities of phyA in the Pr and Pfr forms for downstream signaling components such as PIF1 and PIF3 may be sufficient to inhibit the induction of a VLFR in the dark.

Despite having a low total level of phyA (only around 25% of wild-type levels) inhibition of hypocotyl elongation and promotion of anthocyanin accumulation is very efficiently complemented in the phyA-NLS and phyA-NLS-GFP lines (Figure 3 and S1). These results suggest that nuclear phyA abundance (rather than total phyA levels) primarily controls these light responses. The strong phenotype of the *fhy1 fhl* and *fhy3 far1* double mutants, which do not contain detectable levels of phyA in the nucleus, further supports this view [26],[31],[33]. Thus, nuclear accumulation of both phyA and phyB has been shown to be functionally important in Arabidopsis [26],[27],[29]. While these studies show that this is an important step of the signal transduction cascade for numerous phytochrome responses, they by no means exclude the possibility for cytoplasmic activities of the phytochromes. Cytoplasmic phytochrome responses are widely described in cryptogam species [45]–[47] and a recent paper indicates that cytoplasmic phyA may be required for the modulation of the phototropic response in Arabidopsis [11].

The vast majority of proteins enters the nucleus either passively or by active, importin-mediated transport [35]. However, there are nuclear localized proteins, which are too big to pass through the nuclear pore by diffusion but still do not contain an NLS. Similar to phyA many of these proteins use a piggyback mechanism and rely on the NLS of an interacting protein for nuclear transport [48]–[56]. Yet, in contrast to phyA, most of these proteins seem to interact with the NLS containing protein constitutively [48],[49],[51],[52],[56] or they are even part of a stable oligomeric complex with one of its components providing an NLS [53],[55]. Often the NLS containing protein also performs an essential function besides nuclear transport [49]–[52],[54]. Compared to the piggyback systems described above, the FHY1/phyA system is unique inasmuch as i) nuclear transport of the cargo protein is regulated by a conformational change of phyA [27] and ii) the NLS containing protein is dedicated exclusively to nuclear transport of the cargo protein given that FHY1 becomes dispensable in a strain where phyA possesses it own NLS (Figure 5).

## Materials and Methods

### Constructs, Transgenic Plants

To obtain the *P_PHYA_*∶*PHYA*-*NLS*-*GFP5* construct (CF461), we inserted the following sequence AALQKKKRKVGGAAA between phyA and GFP5 of CF161 [27] using standard molecular biology techniques (NLS is underlined). *P_PHYA_*∶*PHYA*-*NLS* (CF460) is the same construct except that there is a stop codon directly after the last codon of the NLS sequence (i.e. does not contain GFP5).

Transgenic plants expressing phyA-NLS (CF460) and phyA-NLS-GFP (CF461) under the control of the *PHYA* promoter were obtained by transforming the constructs (CF460, CF461) into *phyA*-*211* mutants by *Agrobacterium*-mediated transformation [57]. Transgenic plants were selected on 0.5× Murashige & Skoog (MS) medium (Duchefa), 0.7% agar (Sigma) with 30 µg/ml kanamycin. Single insertion lines were selected by determining the *kan^r^*/*kan^s^* ratio in T2. Homozygous progeny of two representative single insertion lines for each construct were used for further studies.

pphyA40-phyA (contains *P_PHYA_*∶*PHYA*-*CFP*∶*Ter_RbcS_*) is a T-DNA vector derived from pCHF40-phyA (contains *P_35S_*∶*PHYA*-*CFP*∶*Ter_RbcS_*) and was used to generate plants expressing *PHYA* promoter driven phyA-CFP. pphyA40-phyA and pCHF40-phyA were obtained as described for pphyA30-phyA and pCHF30-phyA but contain ECFP (Clontech) instead of EYFP [26].

pCHF70-, pCHF72- and pCHF73-FHY1 167–202 are T-DNA vectors used to generate plants expressing CaMV 35S promoter driven YFP-FHY1 CT, NLS-YFP-FHY1 CT (artificial FHY1) and NES-YFP-FHY1 CT. Details regarding cloning of these constructs can be found in Text S1.

pCHF70-, pCHF72- and pCHF73-FHY1 167–202 were used for *Agrobacterium*-mediated transformation of L*er* and *fhy1*-*1* (only pCHF72-FHY1 167–202), pphyA40-phyA for transformation of *phyA*-*201* [57]. Transgenic plants were selected on soil using BASTA (AgrEvo). Unless indicated otherwise, homozygous progeny of single insertion lines (1∶3 segregation of the selection marker) were used for the experiments.

Lines co-expressing either NLS- or NES-YFP-FHY1 CT and phyA-CFP were obtained by genetic crossing of L*er P_35S_*∶*NLS*/*NES*-*YFP*-*FHY1 CT* and *phyA*-*201 P_PHYA_*∶*PHYA*-*CFP* (L*er* ecotype). The F1 generation was used for microscopic analysis.

The *phyA*-*211 fhy1*-*1* plants expressing phyA-NLS-GFP were obtained by crossing *phyA*-*211 P_PHYA_*∶*PHYA*-*NLS*-*GFP5* (Col ecotype) into *fhy1*-*1* (L*er* ecotype) background. In F2 siblings were selected that were homozygous for the transgene and *phyA*-*211* and either wild-type (i. e. *phyA*-*211 FHY1 P_PHYA_*∶*PHYA*-*NLS*-*GFP5*, in Col×L*er* background) or mutant (i. e. *phyA*-*211 fhy1*-*1 P_PHYA_*∶*PHYA*-*NLS*-*GFP5*, in Col×L*er* background) for *FHY1*. In all experiments with *phyA*-*211 fhy1*-*1 P_PHYA_*∶*PHYA*-*GFP5* the *phyA*-*211 FHY1 P_PHYA_*∶*PHYA*-*NLS*-*GFP5* in Col×L*er* background was used as wild-type control.

*phyA*-*211 fhy3*-*1 P_PHYA_*∶*PHYA*-*NLS*-*GFP* plants were obtained by crossing *phyA*-*211 P_PHYA_*∶*PHYA*-*NLS*-*GFP* (Col ecotype) into *fhy3*-*1* (Col ecotype) background. In F2 seedlings homozygous for *phyA*-*211*, *fhy3*-*1* and the transgene were selected.

### Plant Material

The Columbia (Col-0) and Landsberg *erecta* (L*er*) ecotype of *A. thaliana* were used as wild type. *phyA*-*211* [58], *cop1*-*4* [59] and *fhy3*-*1* [60],[61] are in Col while *fhy1*-*1* [61],[62] and *phyA*-*201* [58] are in L*er*. *phyA*-*211 P_PHYA_*∶*PHYA*-*GFP5* (A-GFP1), *phyA*-*211 fhy1*-*1 P_PHYA_*∶*PHYA*-*GFP5*, *phyA*-*211 fhy3*-*1 P_PHYA_*∶*PHYA*-*GFP5* and *fhy1*-*1 P_35S_*∶*YFP*-*FHY1* were previously described [27]. A second *phyA*-*211 P_PHYA_*∶*PHYA*-*GFP5* line (A-GFP2) which was obtained during the screen described previously [27] was used because its phyA-GFP protein level is close to the phyA-NLS-GFP protein level in the lines we obtained.

### Hypocotyl Length, Anthocyanin Accumulation

Measurements of hypocotyl length in continuous FR light and anthocyanin accumulation were performed as described [63]. For hypocotyl length seedlings were grown on half-strength MS, 0.7% agar while for anthocyanin accumulation seedlings were grown on half-strength MS, 0.7% agar supplemented with 1.5% sucrose. The VLFR of hypocotyl elongation and its transition to the HIR was investigated essentially as described [64]. Briefly, chilled seeds were exposed to red light for 6 h followed by 18 h of incubation in darkness before transfer to pulses of FR (3 min) given at different dark intervals (117 min, 57 min, 27 min or 0 min = continuous FR). Hypocotyl length was measured to the nearest 0.5 mm after 3 d of treatment and is expressed relative to dark controls. Data are means and SE of at least 11 replicate boxes (10 seedlings per box).

### Microscopy

Microscopic analyses in Figures 2A–D and 4A–D were performed with a Leica DM 600B equipped with Leica LTR6000 laser (software LAS, Leica Application Suite) using GFP and DAPI filter sets and a 20× air objective. 3-day-old dark-grown seedlings were directly observed under the microscope (dark condition). For light conditions, 3 day-old-dark-grown seedlings were pretreated for 10 min with white light before they were observed under the microscope.

For microscopic analyses in Figures 1C and 1D, 2E and 2F, 4E–H and 7B a Zeiss Axioscope 2 equipped with a 63× oil-immersion objective and GFP, YFP and CFP specific filter sets was used. The seedlings used for microscopy were grown as described in the figure legends.

Materials and Methods for Figures S1–S6 can be found in Text S1.

## Supporting Information

Figure S1 phyA protein levels in our transgenic lines. Col, *phyA*-*211* as well as *phyA*-*211* seedlings expressing *P_PHYA_*∶*PHYA*-*NLS*, *P_PHYA_*∶*PHYA*-*NLS*-*GFP* or *P_PHYA_*∶*PHYA*-*GFP* (i.e. the lines used in this study) were grown in the dark. After 4 days total protein was extracted and separated by SDS-PAGE. Quantitative western blot analysis was used to measure the phyA levels. The mean value +/− SEM of biological triplicates is indicated.
(0.05 MB TIF)

Figure S2 Cytoplasmically localized FHY1 CT induces a dominant negative phenotype. (A) Morphology of seedlings expressing FHY1 CT. Wild-type (L*er*), *fhy1*-*1* and *phyA*-*201* seedlings as well as transgenic lines expressing different FHY1 167–202 ( = FHY1 CT) constructs were grown for 5 days in the dark or in far-red light (15 µmol m^−2^ s^−1^). #2590, #2607, #2619, #2638, #2643; L*er P_35S_*∶*YFP*-*FHY1 167*–*202* (L*er YF CT*). #4520, #4527; L*er P_35S_*∶*NLS*-*YFP*-*FHY1 167*–*202* (L*er NLS*-*YF CT*). #4578, #4597; L*er P_35S_*∶*NES*-*YFP*-*FHY1 167*–*202* (L*er NES*-*YF CT*). (B) Protein levels in seedlings expressing FHY1 CT. Wild-type (L*er*), *fhy1*-*1* and *phyA*-*201* seedlings as well as the transgenic lines shown in (A) were grown for 4 days in the dark. Total protein was extracted and analyzed by SDS-PAGE and immunoblotting. phyA and (NLS-/NES-) YFP-FHY1 CT were detected using polyclonal antibodies specific for the N-terminal half of Arabidopsis phyA and GFP, respectively. The amido black stained PVDF membranes are shown as loading controls (15 µg total protein per lane).
(1.39 MB TIF)

Figure S3 FHY1 interacts with importin alpha. (A) Pull down experiment for FHY1 and importin alpha. *In vitro* synthesized 35S-labeled importin alpha was incubated for 2 hours with recombinant GST-FHY1-H_6_, GST-FHY1 ΔNLS-H_6_ and GST-H_6_ (nonbinding control) bound to GSH sepharose. After washing, the sepharose beads were incubated with SDS-PAGE sample buffer for elution. The samples were separated by SDS-PAGE and transferred onto a PVDF membrane. A phosphorimager was used for signal detection. Lane 1 contains 4% of the input used in lanes 2–4. Both the autoradiogram (top) and the Amido Black-stained membrane are shown. (B) FHY1 ΔNLS normally interacts with phyA. Yeast (strain AH109) was transformed with the indicated plasmids. A 5 µl aliquot of overnight cultures was spotted onto selective synthetic dropout plates (L–W–H–, containing 1 mM 3-aminotriazole) supplemented with 10 µM PCB. The plates were incubated for 3 d in 1 µmol m^−2^ s^−1^ red light (Pfr) or 13 µmol m^−2^ s^−1^ far-red light (Pr). As a control, equal amounts of overnight cultures were spotted onto non-selective (L–W–) plates without PCB. AD, GAL4 activation domain; BD, GAL4 DNA-binding domain.
(0.4 MB TIF)

Figure S4 FHY1 does not protect phyA-NLS-GFP from degradation in the nucleus. (A) Total protein extracts were prepared from seedlings expressing phyA-NLS-GFP in wild-type (*FHY1*) or *fhy1* mutant background. The seedlings were grown for 4 days in the dark (Dark) or irradiated for 1 day with far-red light (15 µmol m^−2^ s^−1^) after 3 days in the dark (FR). The protein extracts were separated by SDS-PAGE and used for immunoblotting with antibodies specific for phyA or DET3 (loading control). (B) phyA-NLS-GFP levels in *FHY1* and *fhy1*-*1* background were quantified using quantitative western blot analysis. The seedlings were grown as described in (A) and the mean value +/− SEM of biological triplicates is indicated. *FHY1* phyA-NLS-GFP; *phyA*-*211 FHY1 P_PHYA_*∶*PHYA*-*NLS*-*GFP* (Col×L*er*). *fhy1* phyA-NLS-GFP; *phyA*-*211 fhy1*-*1 P_PHYA_*∶*PHYA*-*NLS*-*GFP* (Col×L*er*).
(0.11 MB TIF)

Figure S5 Reversible interaction of FHY1/FHL and phyA. (A) explains how the yeast two hybrid β-galactosidase activity assay in (B) was done. (B) Yeast (strain Y187) was transformed with plasmids encoding AD-FHY1/phyA-BD (FHY1) or AD-FHL/phyA-BD (FHL). Overnight cultures supplemented with 10 µM PCB were grown in nonselective medium in the dark. The cultures were then irradiated for 5 min with 12 µmol m^−2^ s^−1^ red light and incubated in the dark for another 240 min before measuring the β-galactosidase activity. Immediately (0 min), 60 min, 120 min or 240 min after the red light pulse a 5 min far-red light pulse (13 µmol m^−2^ s^−1^) was given. Error bars indicate the SEM (n = 3). MU, Miller units.
(0.01 MB TIF)

Figure S6 Nuclear import model explaining FHY1 dependent phyA nuclear accumulation in far-red light. In seedlings irradiated with FR only a minor fraction of the phyA molecules is in the active Pfr from (≤∼2%). Upon binding of PfrA to FHY1 the PfrA-FHY1 complex is transported into the nucleus using the NLS of FHY1 and the general nuclear import machinery. Once in the nucleus most of the transported PfrA-FHY1 complexes will dissociate in FR into PrA and free FHY1. Free FHY1 will recycle to the cytosol and be available for further import cycles. In contrast, PrA and PfrA are trapped in the nucleus because they are i) too big to exit the nucleus by diffusion and ii) not actively exported into the cytosol. How FHY1 recycling works and if dissociation of the phyA-FHY1 complex is essential for initiation of downstream signaling remains unknown.
(0.58 MB TIF)

Table S1 List of accession numbers. The table shows the accession numbers of the sequences used for the alignment in Figure 1A as well as the databases, in which the sequences were found. GenBank (NCBI): http://www.ncbi.nlm.nih.gov/sites/entrezdbnucleotide. JGI (Joint Genome Institute): http://genome.jgi-psf.org/Poptr1_1/Poptr1_1.home.html. MAtDB v2.0 (Arabidopsis Genome Database): http://mips.gsf.de/proj/plant/jsf/athal/. The Gene Index Project: http://biocomp.dfci.harvard.edu/tgi/plant.html.
(0.04 MB DOC)

Text S1 Experimental procedures.
(0.03 MB DOC)
