# Supplementary figures and images for: FHY1 Mediates Nuclear Import of the Light-Activated Phytochrome A Photoreceptor
Source: PLoS Genet. 2008 Aug 1;4(8):e1000143. doi: 10.1371/journal.pgen.1000143 (PMC2483295; doi:10.1371/journal.pgen.1000143)

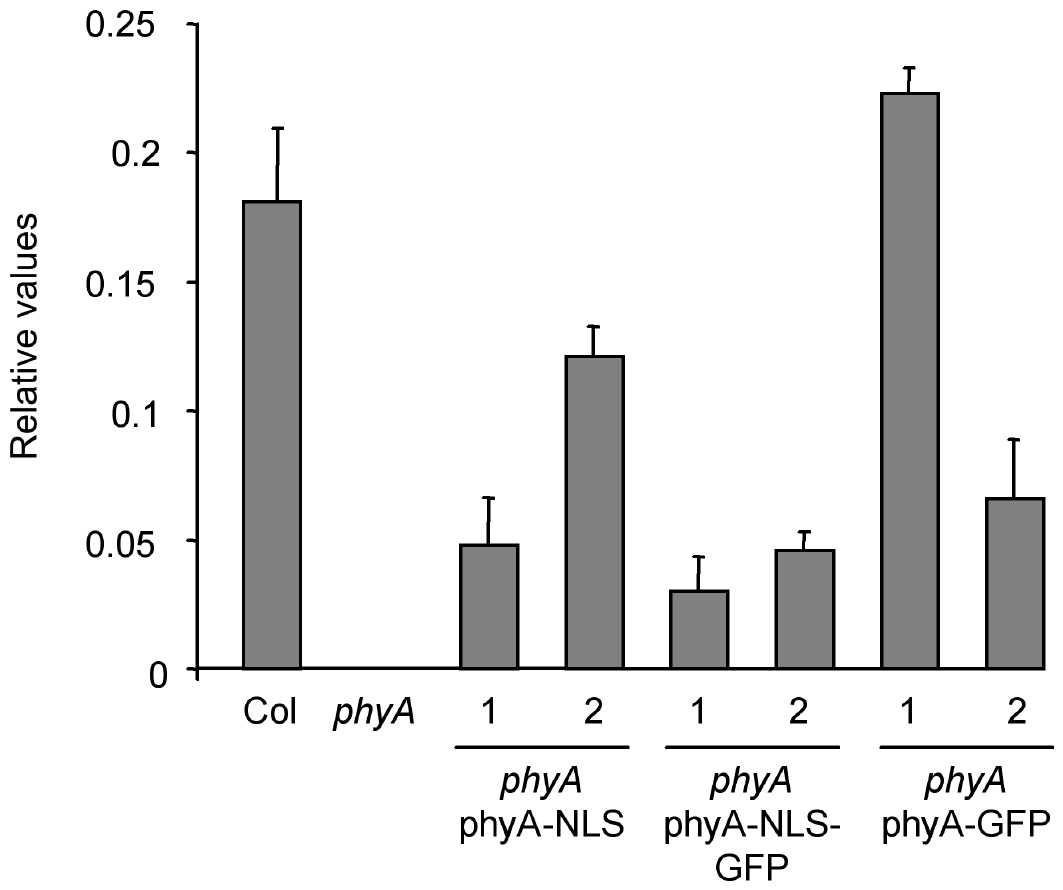

Supplement: Figure S1 — phyA protein levels in our transgenic lines. Col, phyA-211 as well as phyA-211 seedlings expressing PPHYA∶PHYA-NLS, PPHYA∶PHYA-NLS-GFP or PPHYA∶PHYA-GFP (i.e. the lines used in this study) were grown in the dark. After 4 days total protein was extracted and separated by SDS-PAGE. Quantitative western blot analysis was used to measure the phyA levels. The mean value +/− SEM of biological triplicates is indicated. (0.05 MB TIF) [file pgen.1000143.s001.tif]

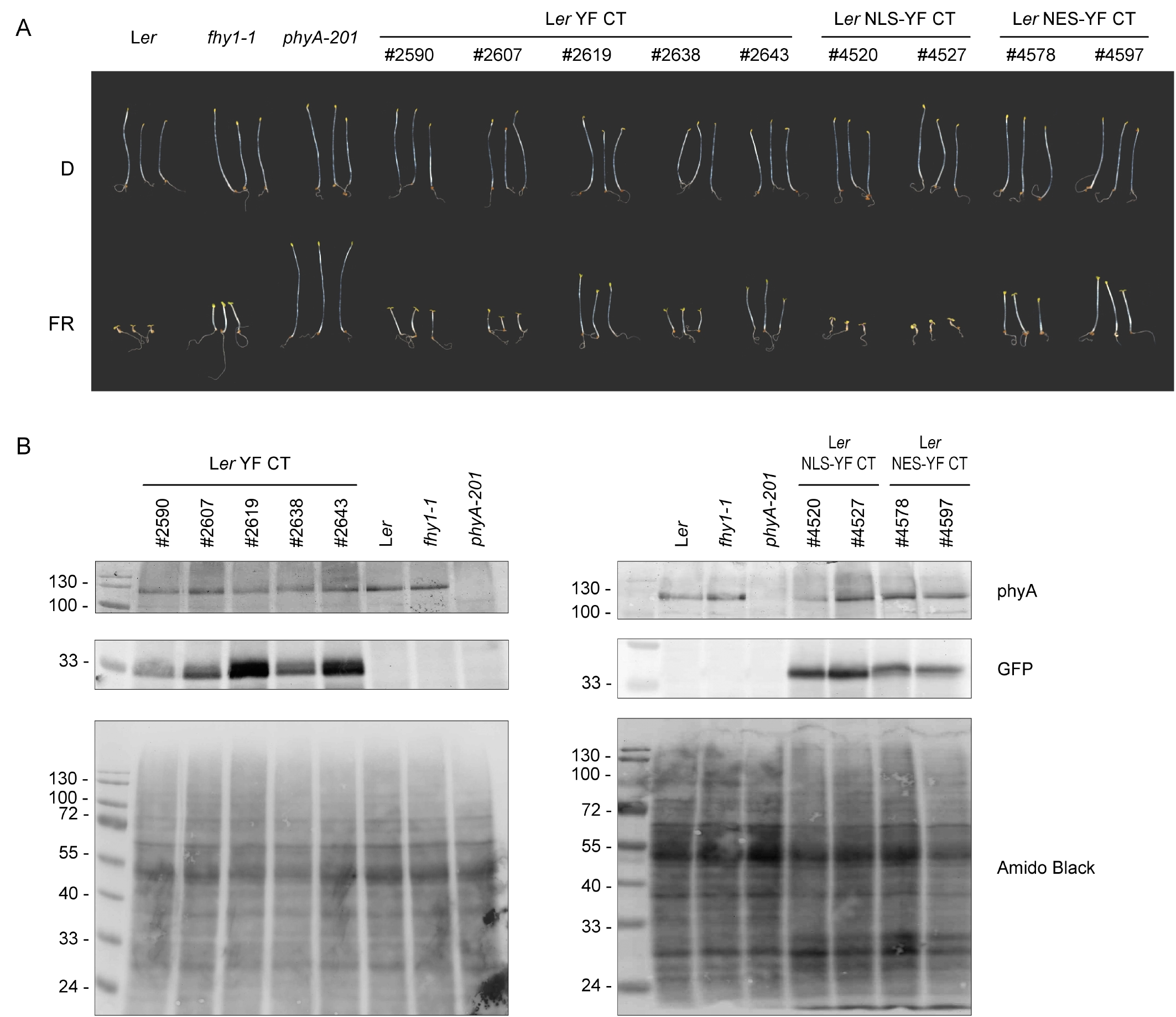

Supplement: Figure S2 — Cytoplasmically localized FHY1 CT induces a dominant negative phenotype. (A) Morphology of seedlings expressing FHY1 CT. Wild-type (Ler), fhy1-1 and phyA-201 seedlings as well as transgenic lines expressing different FHY1 167–202 ( = FHY1 CT) constructs were grown for 5 days in the dark or in far-red light (15 µmol m−2 s−1). #2590, #2607, #2619, #2638, #2643; Ler P35S∶YFP-FHY1 167–202 (Ler YF CT). #4520, #4527; Ler P35S∶NLS-YFP-FHY1 167–202 (Ler NLS-YF CT). #4578, #4597; Ler P35S∶NES-YFP-FHY1 167–202 (Ler NES-YF CT). (B) Protein levels in seedlings expressing FHY1 CT. Wild-type (Ler), fhy1-1 and phyA-201 seedlings as well as the transgenic lines shown in (A) were grown for 4 days in the dark. Total protein was extracted and analyzed by SDS-PAGE and immunoblotting. phyA and (NLS-/NES-) YFP-FHY1 CT were detected using polyclonal antibodies specific for the N-terminal half of Arabidopsis phyA and GFP, respectively. The amido black stained PVDF membranes are shown as loading controls (15 µg total protein per lane). (1.39 MB TIF) [file pgen.1000143.s002.tif]

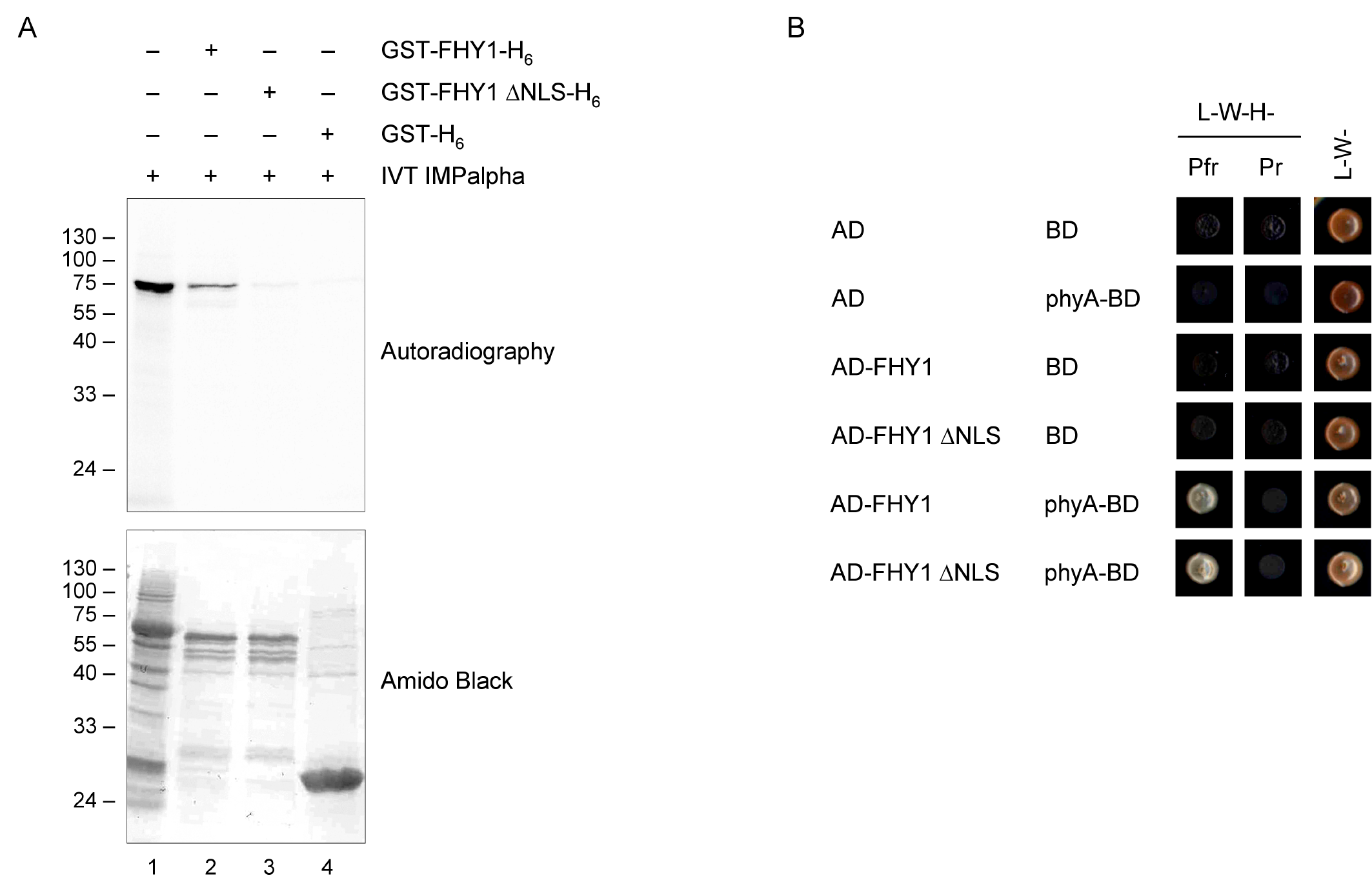

Supplement: Figure S3 — FHY1 interacts with importin alpha. (A) Pull down experiment for FHY1 and importin alpha. In vitro synthesized 35S-labeled importin alpha was incubated for 2 hours with recombinant GST-FHY1-H6, GST-FHY1 ΔNLS-H6 and GST-H6 (nonbinding control) bound to GSH sepharose. After washing, the sepharose beads were incubated with SDS-PAGE sample buffer for elution. The samples were separated by SDS-PAGE and transferred onto a PVDF membrane. A phosphorimager was used for signal detection. Lane 1 contains 4% of the input used in lanes 2–4. Both the autoradiogram (top) and the Amido Black-stained membrane are shown. (B) FHY1 ΔNLS normally interacts with phyA. Yeast (strain AH109) was transformed with the indicated plasmids. A 5 µl aliquot of overnight cultures was spotted onto selective synthetic dropout plates (L–W–H–, containing 1 mM 3-aminotriazole) supplemented with 10 µM PCB. The plates were incubated for 3 d in 1 µmol m−2 s−1 red light (Pfr) or 13 µmol m−2 s−1 far-red light (Pr). As a control, equal amounts of overnight cultures were spotted onto non-selective (L–W–) plates without PCB. AD, GAL4 activation domain; BD, GAL4 DNA-binding domain. (0.4 MB TIF) [file pgen.1000143.s003.tif]

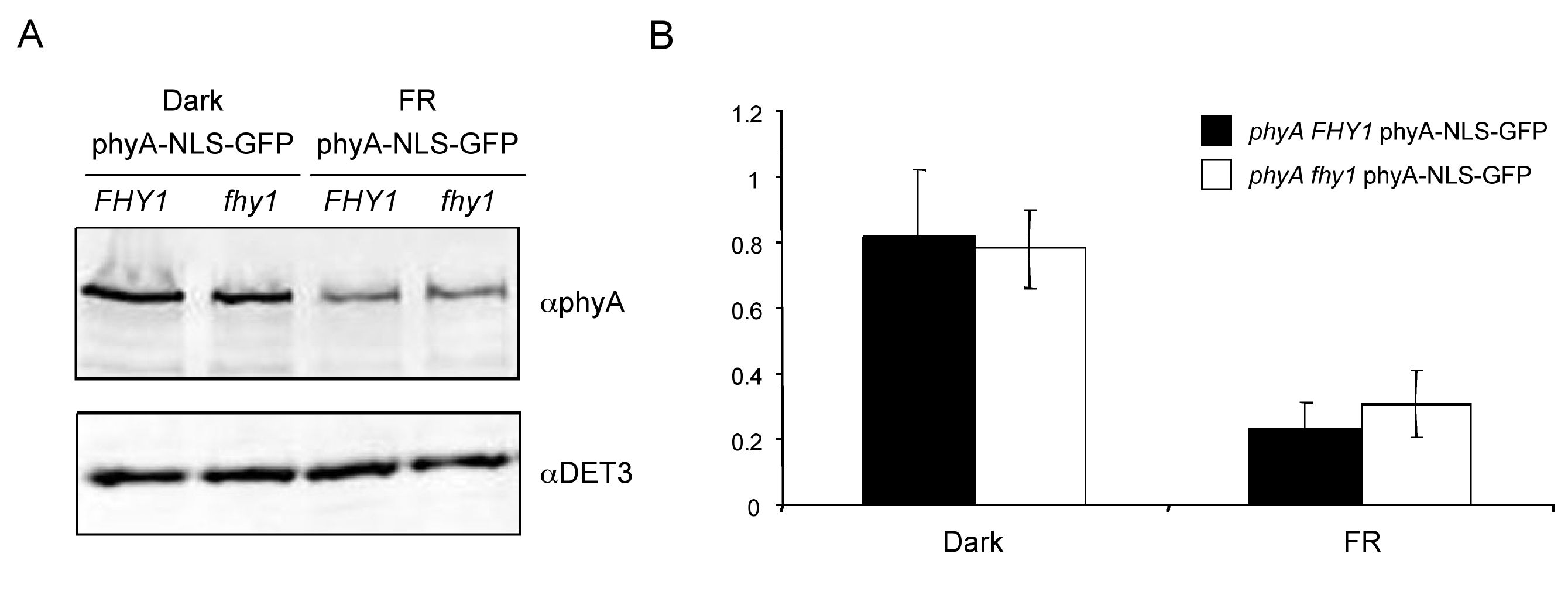

Supplement: Figure S4 — FHY1 does not protect phyA-NLS-GFP from degradation in the nucleus. (A) Total protein extracts were prepared from seedlings expressing phyA-NLS-GFP in wild-type (FHY1) or fhy1 mutant background. The seedlings were grown for 4 days in the dark (Dark) or irradiated for 1 day with far-red light (15 µmol m−2 s−1) after 3 days in the dark (FR). The protein extracts were separated by SDS-PAGE and used for immunoblotting with antibodies specific for phyA or DET3 (loading control). (B) phyA-NLS-GFP levels in FHY1 and fhy1-1 background were quantified using quantitative western blot analysis. The seedlings were grown as described in (A) and the mean value +/− SEM of biological triplicates is indicated. FHY1 phyA-NLS-GFP; phyA-211 FHY1 PPHYA∶PHYA-NLS-GFP (Col×Ler). fhy1 phyA-NLS-GFP; phyA-211 fhy1-1 PPHYA∶PHYA-NLS-GFP (Col×Ler). (0.11 MB TIF) [file pgen.1000143.s004.jpg]

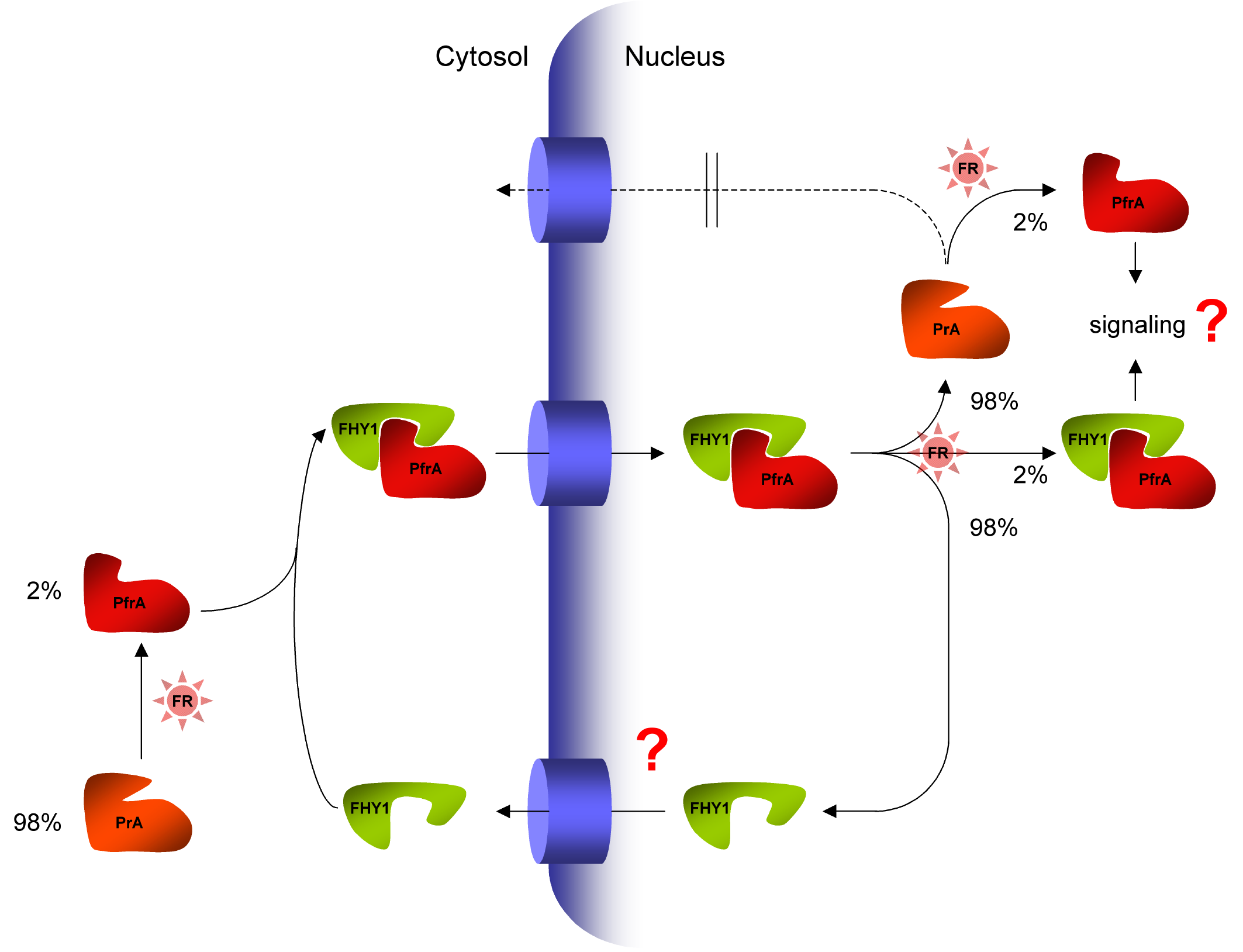

Supplement: Figure S6 — Nuclear import model explaining FHY1 dependent phyA nuclear accumulation in far-red light. In seedlings irradiated with FR only a minor fraction of the phyA molecules is in the active Pfr from (≤∼2%). Upon binding of PfrA to FHY1 the PfrA-FHY1 complex is transported into the nucleus using the NLS of FHY1 and the general nuclear import machinery. Once in the nucleus most of the transported PfrA-FHY1 complexes will dissociate in FR into PrA and free FHY1. Free FHY1 will recycle to the cytosol and be available for further import cycles. In contrast, PrA and PfrA are trapped in the nucleus because they are i) too big to exit the nucleus by diffusion and ii) not actively exported into the cytosol. How FHY1 recycling works and if dissociation of the phyA-FHY1 complex is essential for initiation of downstream signaling remains unknown. (0.58 MB TIF) [file pgen.1000143.s006.tif]
