# Supplementary material for: FHY1 Mediates Nuclear Import of the Light-Activated Phytochrome A Photoreceptor
Source: PLoS Genet. 2008 Aug 1;4(8):e1000143. doi: 10.1371/journal.pgen.1000143 (PMC2483295; doi:10.1371/journal.pgen.1000143)
Supplement: Table S1 — List of accession numbers. The table shows the accession numbers of the sequences used for the alignment in Figure 1A as well as the databases, in which the sequences were found. GenBank (NCBI): http://www.ncbi.nlm.nih.gov/sites/entrezdbnucleotide. JGI (Joint Genome Institute): http://genome.jgi-psf.org/Poptr1_1/Poptr1_1.home.html. MAtDB v2.0 (Arabidopsis Genome Database): http://mips.gsf.de/proj/plant/jsf/athal/. The Gene Index Project: http://biocomp.dfci.harvard.edu/tgi/plant.html. (0.04 MB DOC) [file pgen.1000143.s007.doc]

| **Plant species** | **Accession number(s)** | **Database(s)** |
| --- | --- | --- |
| Ananas comosus | CO731885 | GenBank |
| Aquilegia formosa x pubescens | DR915851, DR915850, DT753904, DR949908, DT756939, DT733705, DR944577, DT741253, DT756938, DT759307, DT737897, DT751471, DR944576, DT753903, DT741252, DR949907 | GenBank |
| Arabidopsis thaliana FHL Col | At5g02200 | MAtDB v2.0 |
| Arabidopsis thaliana FHY1 Col | At2g37675 | MAtDB v2.0 |
| Arachis hypogaea | EE125801, ES764565 | GenBank |
| Aristolochia fimbriata | FD760883 | GenBank |
| Artemisia annua | EY034625, EY034626 | GenBank |
| Avena sativa | CN820303, CN819803 | GenBank |
| Brassica oleracea 1 | AM057896, AM385317, AM388490 | GenBank |
| Brassica oleracea 2 | EH425510, BZ481933 | GenBank |
| Brassica rapa 1 | AC189307 | GenBank |
| Brassica rapa 2 | EX132261, EX125103, EX128646, EX044305 | GenBank |
| Brassica rapa 3 | EX120808 | GenBank |
| Carthamus tinctorius | EL405405 | GenBank |
| Centaurea solstitialis | EH777501 | GenBank |
| Cichorium intybus | EH708694 | GenBank |
| Cucumis melo | AM715072, AM722656 | GenBank |
| Cyclamen persicum | AJ887114 | GenBank |
| Eucalyptus gunnii 1 | CT984070 | GenBank |
| Eucalyptus gunnii 2 | CU397308 | GenBank |
| Euphorbia esula | DV126999, DV122544, DV126897, DV156847, DV155720 | GenBank |
| Glycine max | EV280715, BQ299056, EV278954, EV266329, EV272122, AW569577 | GenBank |
| Gossypium hirsutum | DW491224, DW491223, DN817138, DW488461, DW488460, DN799884, DW515229, DV849522 | GenBank |
| Helianthus paradoxus | EL488842 | GenBank |
| Helianthus petiolaris | DY954681 | GenBank |
| Helianthus tuberosus | EL466034 | GenBank |
| Ipomoea nil | CJ756095, CJ756116, CJ737499, CJ737522 | GenBank |
| Lycopersicum esculentum | TC173848 | The Gene Index Project |
| Malus x domestica | CV986962 | GenBank |
| Manihot esculenta | DV452662 | GenBank |
| Medicago truncatula | BI308149 | GenBank |
| Mesembryanthemum crystallinum | CA835599 | GenBank |
| Musa acuminata | ES433417 | GenBank |
| Nicotiana tabaccum | DW000397 | GenBank |
| Oryza sativa | AK070454 | GenBank |
| Petunia x hybrida | CV293132 | GenBank |
| Picea-Pinus | DR466440, CO239186, CO208568, DR564420, CO484941, CO232975, CK435440, DR557304, DT635653, DR689460 | GenBank |
| Polygonum sibiricum | ES605106 | GenBank |
| Populus euramericana x canadensis | CX178123 | GenBank |
| Populus nigra | DB903192 | GenBank |
| Populus tremula | BU866661, CK097822 | GenBank |
| Populus tremula x tremuloides | BU895669 | GenBank |
| Populus trichocarpa 1 | eugene3.00060737 | JGI |
| Populus trichocarpa 2 | eugene3.00160946 | JGI |
| Prunus persica | AJ826744, DY637329, DY633702 | GenBank |
| Pseudotsuga menziesii | ES424557 | GenBank |
| Raphanus raphanistrum 1 | EY909567, EY909639, EY913312, EY913402, FD958999, FD977120, FD981342 | GenBank |
| Raphanus raphanistrum 2 | EY907892, EY903716 | GenBank |
| Raphanus sativus 1 | EX898049, EX901688 | GenBank |
| Raphanus sativus 2 | FD946136, FD942321 | GenBank |
| Saccharum officinarum | TC52633, CA233075 | The Gene Index Project, GenBank |
| Salvia miltiorrhiza | CV169421 | GenBank |
| Saruma henryi | DT582798 | GenBank |
| Solanum tuberosum | CK276046 | GenBank |
| Sorghum bicolor | CF073214 | GenBank |
| Taraxacum officinale | DY831102 | GenBank |
| Trifolium pratense | BB914683 | GenBank |
| Triphysaria pusilla | EY183043, EY175781, EY175780 | GenBank |
| Triticum aestivum | TC237408 | The Gene Index Project |
| Vitis vinifera | EC952348 | GenBank |
| Zea mays | TC321138 | The Gene Index Project |
| Zingiber officinale | DY375058 | GenBank |
