# Supplementary material for: FHY1 Mediates Nuclear Import of the Light-Activated Phytochrome A Photoreceptor
Source: PLoS Genet. 2008 Aug 1;4(8):e1000143. doi: 10.1371/journal.pgen.1000143 (PMC2483295; doi:10.1371/journal.pgen.1000143)
Supplement: Text S1 — Experimental procedures. (0.03 MB DOC) [file pgen.1000143.s008.doc]

# **Text S1**

**FHY1 mediates nuclear import of the light-activated phytochrome A photoreceptor.**

Thierry Genoud ^1^, Fabian Schweizer ^1^, Anke Tscheuschler ^2^, Dimitry Debrieux ^1^, Jorge J. Casal ^3^, Eberhard Schäfer ^2, 4^, Andreas Hiltbrunner ^2, 4, 5^ and Christian Fankhauser ^1, 5^

^1^ Centre for Integrative Genomics, University of Lausanne, Genopode Building, CH‑1015 Lausanne, Switzerland.

^2^ Institut für Biologie II/Botanik, Albert Ludwigs Universität, Schänzlestrasse 1, D‑79104 Freiburg, Germany.

^3^ IFEVA, Facultad de Agronomía, Universidad de Buenos Aires, and Consejo Nacional de Investigaciones Científicas y Técnicas (CONICET), Av. San Martin 4453, 1417-Buenos Aires, Argentina

^4^ BIOSS, Centre for Biological Signalling Studies, University of Freiburg, Albertstrasse 19, D-79104 Freiburg, Germany

^5^ Corresponding authors:

Christian Fankhauser Andreas Hiltbrunner

christian.fankhauser@unil.ch andreas.hiltbrunner@biologie.uni-freiburg.de

phone: ++41 21 692 3941 phone: ++49 761 203 2807

FAX: ++41 21 692 3925 FAX: ++49 761 203 2612

**Supplementary Materials and Methods**

Constructs

pCHF72 and pCHF73 are T-DNA vectors containing *P_35S_:NLS-YFP-BamHI-XbaI:Ter_RbcS_* and *P_35S_:NES-YFP-BamHI-XbaI:Ter_RbcS_* cassettes, respectively. To obtain them YFP was amplified from pEYFP (Clontech) using a 5’ primer including either the NLS [1] or NES [2] motif as well as a *BglII* site (5’ primer containing NLS: 5’- gaa GAT CTA AAA ATG GAT AAA GCG GAA TTA ATT CCC GAG CCT CCA AAA AAG AAG AGA AAG GTC GAA TTG GGT ACg GCg ATG GTG AGC AAG GGC GAG-3’; 5’ primer containing NES: 5’-gaa gat cta aaa atg CTG CAa AAC GAG CTT GCT CTT AAG TTG GCT GGA CTT GAT ATT AAC AAG ACT GGA GGA gtg GAT CTA AAA ATG GTG AGC AAG GGC GAG-3’). The 3’ primer (5’-gga cta gtt atc tag agc CCT AGG ATC Cgc CTT GTA CAG CTC GTC CAT G-3’) contained *BamHI*, *AvrII*, *XbaI* and *SpeI* sites. The PCR products were digested with *BglII*/*SpeI* and ligated into pCHF5 [3] cut with *BamHI*/*XbaI* resulting in pCHF72 and pCHF73.

To obtain pCHF70 [3]-, pCHF72- and pCHF73-FHY1 167-202 (encoding YFP-FHY1 CT, NLS-YFP-FHY1 CT = “artificial FHY1” and NES-YFP-FHY1 CT) FHY1 167-202 was cut from pGADT7ah-FHY1 167-202 [4] using *BamHI*/*SpeI* and ligated into the *BamHI*/*XbaI* sites of pCHF70, pCHF72 and pCHF73.

The FHY1 ∆NLS constructs used for yeast two hybrid (pGADT7ah-FHY1 ∆NLS) and pull down assays (pGEX6P1-FHY1 ∆NLS-H_6_) were obtained as follows: The N- and C-terminal halves of FHY1 were PCR amplified from pBS II KS-FHY1 [3] using primers including suitable restriction sites as well as the nucleotides coding for the mutated NLS (N-terminal half forward: 5’-cgc GGA TCC AAA AAT GCC TGA AGT GGA AGT G-3’, contains a *BamHI* site; N-terminal half reverse: 5’-gct cta gag cgA gCT CAT CAG ATT GAT CTG TCT GAA AgT TCC TaT TCT TGC TCA CCT CA-3’, contains the ∆NLS mutation as well as *XbaI* and *SacI* sites; C-terminal half forward: 5’-cgc gga tcc gcG AGc TcT CGT TAT TAC CTC TG-3’, contains *BamHI* and *SacI* sites; C-terminal half reverse: 5’-CCT CTT CGC TAT TAC GCC AG-3’). The PCR products were digested with *BamHI*/*XbaI* (N-terminal half) or *SacI* (C-terminal half) and ligated into pBS II KS-FHY1 cut with *BamHI*/*XbaI* and *SacI*, respectively, resulting in pBS II KS-FHY1 ∆NLS N and pBS II KS-FHY1 ∆NLS C. To obtain pBS II KS-FHY1 ∆NLS the C-terminal half of FHY1 ∆NLS was cut with *SacI* from pBS II KS-FHY1 ∆NLS C and ligated into the *SacI* site of pBS II KS-FHY1 ∆NLS N. Then FHY1 ∆NLS was cut from pBS II KS-FHY1 ∆NLS using *BamHI*/*SpeI* and ligated into the *BamHI*/*SpeI* sites of pGADT7ah-FHY1 and pGEX6P1-FHY1-H_6_ [3] to replace wild type FHY1.

pGADT7ah-IMPα, which was used for *in vitro* transcription/translation of importin alpha, was obtained as follows: importin alpha (At5g06720) was PCR amplified from cDNA clone U21893 (ABRC) using primers containing *BamHI* and *XbaI* sites (5’-cgc GGA TCC AAA AAT GTC ACT GAG ACC CAA CG-3’ and 5’-gct cta gaG CTG AAG TTG AAT CCT CCG-3’). Then the PCR product was cut with *BamHI*/*XbaI* and ligated into pGADT7ah-FHY1 [3] digested with *BamHI*/*SpeI* to replace FHY1.

Yeast two hybrid and pull down assays

Yeast two hybrid growth and β-galactosidase assays were done as described [3] but the PCB concentration was reduced from 20 to 10 µM. pGADT7ah-FHY1, pGADT7ah-FHL and D153ah-phyA (encoding GAL4 AD-FHY1, GAL4 AD-FHL and phyA-GAL4 BD) have been described [4].

pGEX6P1-FHY1-H_6_ and pGEX6P1-H_6_ (encoding GST-FHY1-H_6_ and GST-H_6_) have been described [3]. pGEX6P1-FHY1 ∆NLS-H_6_ as well as pGEX6P1-FHY1-H_6_ and pGEX6P1-H_6_ were transformed into *E. coli* DH5α for protein expression. Expression and purification with Ni-NTA agarose (Qiagen) was done according to the supplier’s instructions. 35S-labeled importin alpha was synthesized in a reticulocyte-based system by the TNT Quick Coupled Transcription/Translation kit (Promega) using pGADT7ah-IMPα as template. To reduce unspecific binding to the resin used in the pull-down assay, 35S-labeled importin alpha was precleared. 25 µl GSH sepharose (Amersham Biosciences) and 75 µl 2x binding buffer (2x PBS supplemented with 0.2% v/v NP-40, 0.1% w/v BSA, and 1 tablet/25 ml Roche complete protease inhibitor (Roche)) were added to 50 µl *in vitro* synthesized importin alpha. After 1 h incubation at 4 ºC, the GSH sepharose beads were pelleted and the supernatant was used as precleared prey. Pull-down assays were done in 1x binding buffer in a total volume of 100 µl and contained 50 µl precleared prey protein and 25 µl bait protein bound to GSH sepharose (roughly 1 µg GST-FHY1-H_6_/GST-FHY1 ∆NLS-H_6_ or 5 µg GST-H_6_). After 2 h incubation at 4 ºC the GSH sepharose beads were washed three times with 1 ml wash buffer (same as binding buffer but without BSA). For elution the GSH sepharose beads were incubated for 10 min in 1x SDS-PAGE sample buffer at 65 ºC. Eluate fractions as well as 2 µl of precleared importin alpha (input) were run on an SDS-PAGE. The gel was blotted onto PVDF membrane, which was stained with Amido Black and exposed to a phosphorimager plate.

Quantitative western blot

Quantitative western blot analysis of phyA was performed as described [5].

**References**

1. Kalderon D, Roberts BL, Richardson WD, Smith AE (1984) A short amino acid sequence able to specify nuclear location. Cell 39: 499-509.

2. Wen W, Meinkoth JL, Tsien RY, Taylor SS (1995) Identification of a signal for rapid export of proteins from the nucleus. Cell 82: 463-473.

3. Hiltbrunner A, Viczian A, Bury E, Tscheuschler A, Kircher S, et al. (2005) Nuclear accumulation of the phytochrome A photoreceptor requires FHY1. Curr Biol 15: 2125-2130.

4. Hiltbrunner A, Tscheuschler A, Viczian A, Kunkel T, Kircher S, et al. (2006) FHY1 and FHL act together to mediate nuclear accumulation of the phytochrome A photoreceptor. Plant Cell Physiol 47: 1023-1034.

5. Trupkin SA, Debrieux D, Hiltbrunner A, Fankhauser C, Casal JJ (2007) The serine-rich N-terminal region of Arabidopsis phytochrome A is required for protein stability. Plant Mol Biol 63: 669-678.
